# Salivary IL-1 Beta Level Associated with Poor Sleep Quality in Children/Adolescents with Autism Spectrum Disorder

**DOI:** 10.3390/pediatric16040081

**Published:** 2024-10-31

**Authors:** Milagros Fuentes-Albero, Mayra Alejandra Mafla-España, José Martínez-Raga, Omar Cauli

**Affiliations:** 1 Clínica Ripalda, 46002 Valencia, Spain; milafuentesalbero@gmail.com; 2 Nursing Department, University of Valencia, 46010 Valencia, Spain; maymaes@alumni.uv.es; 3 Department of Psychiatry and Clinical Psychology, Hospital Universitario Doctor Peset and University of Valencia, 46010 Valencia, Spain; martinezragaj@gmail.com

**Keywords:** cytokines, inflammation, sleep, insomnia, children, biomarker

## Abstract

Background: Sleep disorders are common in youths with autism spectrum disorders. Inflammatory cytokines such as Il-1 beta and Il-6 in saliva have been associated with alterations in sleep quality in various conditions. We assessed whether there were associations between the salivary concentration of IL-1 beta and IL-6 and sleep quality in youths with ASD versus typically developing (TD) age- and gender-matched youths. Method: Forty children and adolescents with ASD or TD participated in this study (20% females). Their parents answered the items of a validated questionnaire on sleep quality (Pittsburgh Sleep Quality Index). Results: The mean Pittsburgh score was significantly higher (i.e., the quality of sleep was poorer) in the ASD group (8.68 ± 0.35 (SEM), ranging from 7 to 12 points), compared to the TD group (7.35 ± 0.54 (SEM), ranging from 2 to 12 points) (*p* = 0.02, Mann–Whitney U test). There were no significant differences in the salivary concentration of Il-1 beta and IL-6 receptor between the two groups, but salivary IL-1 beta concentration was inversely associated with poor sleep quality in the ASD group. No associations between the salivary Il-6 concentration and sleep quality were found in either group. Linear regression analysis by separate groups revealed significant associations between the sleep quality score and the concentration of IL-1 beta in the ASD group (*p* = 0.01, OR = −0.53, 95% CI −0.008–0.001). In contrast, no significant associations were observed in the TD group, or for IL-6 in either group. No significant effects of sex, age, or use of psychotropic medications were found. Conclusions: Children and adolescents with ASD showed significantly poorer sleep quality based on their parents’ reports compared to the TD group, and the salivary IL-1 beta concentration was inversely associated with sleep quality only in the ASD group. Further studies on the associations between inflammatory cytokines and sleep in ASD are needed.

## 1. Introduction

Children and adolescents with autism spectrum disorder (ASD) frequently suffer social interaction and communication problems. Those with ASD often have difficulty with social communication and social behaviors, and interests, hobbies, and mannerisms may exhibit restricted and repetitive patterns. Generally, the prevalence of autism worldwide is reported to be around 1% [1]. However, estimates place the incidence rate across Europe between 0.5% and 0.6% [2,3]. A variety of studies confirm that sleep disorders occur in between 44% and 83% of individuals with ASD regardless of cognitive functioning and the severity of ASD [4,5].

Sleep problems in autism are prominent and multifactorial due to biological, psychological, social, environmental, and familial factors [6,7]. The sleep problems most commonly reported by parents/caregivers include difficulties falling and staying asleep, frequent nighttime awakenings, early awakenings, reduced total sleep time, and resistance to going to bed [8,9,10]. One of the few published follow-up studies on sleep and ASD revealed that children with autism are ten times more likely to experience chronic insomnia than the control group and that sleep-related problems were much more persistent over time, with a remission rate of only 8.3% versus 52.4% for the controls [11].

The etiology of ASD is complex and involves multiple factors, including several biological and environmental factors [12]. The immune system affects brain function in individuals with ASD [13]. Numerous studies have indicated changes in pro-inflammatory cytokines in people with ASD [14,15,16]. Moreover, insomnia also activates the inflammatory cascade with a secretion increase in the C-reactive protein, IL-6, and TNFα [17,18]. Both IL-1 beta and TNFα modulate sleep and the sleep–wake cycle, and low levels of these cytokines are related to insomnia [19]. Elevated levels of IL-6 are linked to excessive daytime sleepiness [20]. Several studies have shown that reduced sleep time increases blood secretion of inflammatory mediators [21,22]. The fact that some pro-inflammatory substances such as the cytokines IL-1 beta, TNFα, IL-6, and INF-y show a circadian pattern, with the highest concentrations at night, suggests a potential role for these molecules in the physiological regulation of sleep in the absence of an immune challenge [23,24]. The relationship between inflammation and sleep appears to be bidirectional [25,26]. In addition, several cytokines and their receptors have been shown to be present and biologically active in the central nervous system of healthy organisms, where they interact physiologically with neuronal circuits associated with sleep regulation (e.g., the serotonergic, GABAergic, and cholinergic systems) [17]. Although the list of cytokines and chemokines that have been shown to affect sleep is extensive, the concentrations of Il-1 beta and IL-6 in biological samples have been associated with alterations in sleep quality in various populations [20,27,28,29,30].

Saliva is a biological fluid that contains mainly water (99%) and 1% organic and inorganic substances, with most of them in equilibrium with their concentration in plasma [31]. Recently, it has been established as a valuable diagnostic system for specific patient groups due to its easy accessibility, low cost, and non-invasive method of collection. Patients who might have trouble with venipuncture would particularly benefit from this [32]. According to the results from a proteomic study, proteins in saliva may help with the early detection of sleep disturbances in children with ASD, thus enabling faster intervention [33].

Among cytokines, concentrations of IL-1 beta and IL-6 in saliva have been reported as being significantly associated with poor sleep quality [22,27]. Although biomarkers of stress and inflammation have traditionally been studied in blood, salivary IL-1β and IL-6 are strongly correlated with blood levels [34,35,36]. After several days of sleep deprivation, IL-1β levels in the blood increase [37].To date, studies on the relationship between the salivary concentration of IL-1β and sleep disorders have not shown increased values for IL-1β [27].

Since poor sleep quality is common in ASD, and due to the proposed association between alteration of inflammatory cytokine in saliva samples in different disorders characterized by poor sleep quality, we hypothesized that salivary cytokines concentration could be associated with sleep quality in ASD. The main objective of this study was to analyze sleep quality in a sample of children and adolescents with ASD compared to a control group of the same age and sex and assess whether there is a relationship between sleep quality and the salivary concentration of Il-1 beta and IL-6.

## 2. Materials and Methods

### 2.1. Participants

This observational case–control study was carried out with a group of children and adolescents diagnosed with ASD who were receiving outpatient care in Child and Adolescent Psychiatry in Valencia, Spain, between 2021 and 2022.

The inclusion criteria in the case group were to have an ASD diagnosis confirmed by the two psychiatrists and co-authors of the study (M.F-A and J.M-R) based on the criteria proposed by the Diagnostic and Statistical Manual of Mental Disorders (DSM-5). One of them, M.F-A, is a child and adolescent psychiatrist with 19 years of experience. Patients with ASD were selected through consultation with M.F-A. The control group was composed of neurotypical children and adolescents who did not present signs of ASD, recruited from two regular public schools in the same city (Valencia, Spain). The control group had to be in the same age group as the cases. The children and adolescents were identified by the principals of the schools, and their parents were contacted by the researchers by phone, asking them to participate in the study. The control group included neurotypical, healthy children and adolescents matched by age and gender (in a 1:1 ratio), and they were recruited from two public schools in Valencia. This matching enhances the efficiency of the estimates when the variables used for matching are related to both the condition being studied and dietary habits [38]. The exclusion criteria in both groups were individuals <3 years of age and ≥16 years of age, having concomitant inflammatory or autoimmune disorders, or receiving immune-suppressive or anti-inflammatory drugs that can alter cytokine production. In both cases, the sampling was not probabilistic. All parents were contacted by telephone, and they were enrolled in the study if they accepted and signed informed consent.

The diagnosis of ASD was validated by a qualified psychiatrist using the DSM-5 diagnostic criteria [39]. During a routine appointment with the child psychiatrist, the parents of the children and adolescents with ASD were interviewed. Additional clinical information, including the ASD diagnosis, medications, comorbid conditions, and anthropometric data, was obtained from the medical records.

The body mass index (BMI) was determined by dividing weight in kilograms by the square of height in meters. For children and adolescents, BMI is specific to age and gender and is commonly referred to as BMI for age. In accordance with international guidelines, BMI was classified into four categories: underweight (BMI below the 5th percentile), healthy weight (BMI at or above the 5th percentile but below the 85th percentile), overweight (BMI equal to or greater than the 85th percentile but less than the 95th percentile), and obese (BMI at or above the 95th percentile) [40,41,42].

The study protocol was approved by the Ethics Research Committee of the University of Valencia (Valencia, Spain) (protocol number H1397475950160). The parents of the children and adolescents participating had to sign a form to give their written informed consent for participation in the study.

### 2.2. Pittsburgh Sleep Quality Index (PSQI)

The Pittsburgh Sleep Quality Index (PSQI), developed by Buysee et al. [43], is a well-known instrument for analyzing sleep quality in the previous month. It has been validated in the Spanish language [44]. The instrument provides an overall score for sleep quality and for seven subdomains: subjective sleep quality, sleep latency, sleep duration, sleep efficiency, sleep disturbances, use of sleep medications, and daytime dysfunction. Each subdomain is scored on a scale of 0 to 3, with a total score ranging from 0 to 21 and a higher score describing poorer sleep quality. A PSQI total score of over 5 has been validated as highly sensitive and specific for distinguishing good sleepers from poor sleepers in various populations [43]. Parents of both children/adolescents with ASD and TD completed the PSQI.

### 2.3. IL-1β and IL-6 Measurement

Saliva samples were collected in the afternoon between 4 and 6 p.m. using the Salivette^®^ device manufactured by Sarstedt to measure the concentrations of IL-1β and IL-6. The parents were instructed to refrain their sons/daughters from eating, drinking, or performing any oral hygiene activities for at least one hour before sample collection. Each sample was then centrifuged to eliminate mucins, insoluble materials, and cellular debris. The resulting liquid was divided into equal portions and stored in Eppendorf tubes, which were frozen at −80 °C for future analysis. ELISA immunoassay analyses were carried out using an IL-1β High-Sensitivity Human ELISA Kit (Ab214025, Abcam, The Netherlands) and an IL-6 High-Sensitivity Human ELISA Kit (Ab178013, Abcam, The Netherlands) following the manufacturer’s instructions.

### 2.4. Statistical Analysis

The continuous variables are expressed as the mean and ± standard error (SEM) range and the categorical variables as frequencies with their percentage. The normal distribution of each variable was assessed with the Shapiro–Wilk test in order to determine whether a parametric or non-parametric test should be applied. In our study, a non-normal distribution of quantitative variables at least in one of the two comparison groups (ASD vs. TD) indicated that non-parametric tests should be used. The correlation between two quantitative variables was determined by Spearman’s correlation test (because of the non-normal data distribution). The non-parametric Mann–Whitney U test was performed to compare the differences between the two groups. Pearson’s chi-square test was applied to examine the statistical association between two categorical variables. Linear regression analysis was performed to examine the relationship between sleep quality and several predictor variables, including age, sex, group (ASD vs. TD), and salivary IL-1β concentration. The statistical significance was set at *p* < 0.05. All the statistical analyses were performed using the SPSS statistical package (version 28.0; SPSS, Inc., Chicago, IL, USA).

## 3. Results

### 3.1. Sample Characteristics

The sample of 40 patients diagnosed with ASD was assessed and compared against typically developing (TD) people. The mean age of the participants was 9.90 ± 0.43 (SEM) and the ages of the participants ranged from 5 to 14 years. The majority of participants were male, constituting around 80% of the sample, while female participants were approximately 20 percent only. Considering the patients included in this study, 12 patients with ASD (60%) took either one or more psychotropic medications such as antiepileptics, antidepressants, antipsychotics, or alpha-2 adrenergic agonists. The other eight patients (40%) had been medication-free for at least 6 months. No child or adolescent in the TD group was reported to be on any psychotropic medications. Based on the level of education, in the ASD group, 14 (35%) were in primary school, 16 (40%) were in secondary school, and 10 (25%) were in special school programs. None of the TD children and adolescents included in this study were on any medication or diagnosed with any other acute or chronic condition.

### 3.2. Sleep Quality in ASD and TD Groups

**Figure 1:**
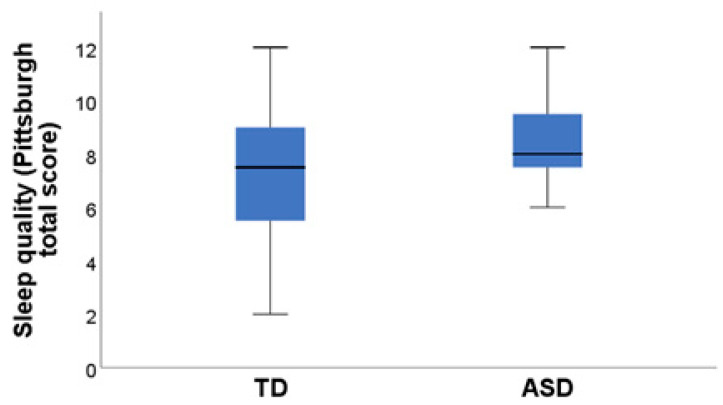
Differences in sleep quality between the ASD group and the TD group.

The mean PSQI score for the ASD group was 8.68 ± 0.35 (SEM) (ranging from 7 to 12 points) and 7.35 ± 0.54 (SEM) (ranging from 2 to 12 points) for the TD group. There were significant differences in sleep quality between the two groups (*p* = 0.02, Pearson’s chi-square test) (Figure 1). A cut-off score of 5 points on the PSQI was used to evaluate sleep quality. In the ASD group, 100% of the participants (20 people) had poor sleep quality compared to 25% of the participants in the TD group showing poor sleep quality (5 people), while 75% (15 people) slept adequately based on the Pittsburgh scale cut-off.Analysis of Pittsburgh Sleep Quality Index Subdomains between ASD and TD group are shown in Table 1.

### 3.3. Relationship Between Sleep Quality and Sociodemographic and Clinical Variables

No significant correlations were found between the PSQI score and age in the overall sample (Rho = −0.11, *p* = 0.48). There were also no significant correlations between the PSQI score and age in the ASD or in the TD group (Rho = −0.28, *p* = 0.23) (Rho = 0.04, *p* = 0.86, Spearman correlation).

No significant correlations were found between the seven subdomains of the PSQI with age in the ASD group: sleep quality (Rho = −0.11, *p* = 0.62), sleep latency (Rho = −0.30, *p* = 0.18), sleep duration (Rho = 0.24, *p* = 0.30), sleep efficiency (Rho = 0.13, *p* = 0.55), sleep disturbances (Rho = −0.43, *p* = 0.05), use of hypnotic medication (Rho = 0.14, *p* = 0.55), and daytime dysfunction (Rho = 0.12, *p* = 0.59; Spearman correlation in all cases).

Likewise, there were no significant correlations between the subdomains of the PSQI and age in the TD group: subjective sleep quality (Rho = −0.02, *p* = 0.90), sleep latency (Rho = −0.03, *p* = 0.88), sleep duration (Rho = −0.20, *p* = 0.38), sleep efficiency (Rho = 0.007, *p* = 0.97), sleep disturbances (Rho = −0.11, *p* = 0.64), use of hypnotic medication (Rho = 0.26, *p* = 0.25), and daytime dysfunction (Rho = 0.22, *p* = 0.33, Spearman correlation in all cases).

There were also no significant differences between the total PSQI score and gender in the overall sample (*p* = 0.44) or the ASD or TD groups (*p* = 0.18 and *p* = 0.97, Mann–Whitney U test in all cases). In addition, there were no significant differences between the seven subdomains of the PSQI and gender in the ASD group: subjective sleep quality (*p* = 0.75), sleep latency (*p* = 0.12), sleep duration (*p* = 0.89), sleep efficiency (*p* = 0.29), sleep disturbances (*p* = 0.06), use of hypnotic medication (*p* = 0.89), and daytime dysfunction (*p* = 0.21, Mann–Whitney U test in all cases).

Similarly, no significant differences were observed between the seven subdomains of the PSQI and gender in the TD control group: subjective sleep quality (*p* = 0.49), sleep latency (*p* = 0.43), sleep duration (*p* = 0.75), sleep efficiency (*p* = 1.00), sleep disturbances (*p* = 0.96), use of hypnotic medication (*p* = 0.82), and daytime dysfunction (*p* = 0.49, Mann–Whitney U test in all cases).

Finally, no significant differences were observed between the total score of the PSQI based on the use of psychotropic medication in ASD (*p* = 0.07, Kruskal–Wallis test).

### 3.4. Relationship Between the Concentrations of IL-1β and IL-6 in Saliva and Sleep Quality in the ASD and TD Groups

**Figure 2:**
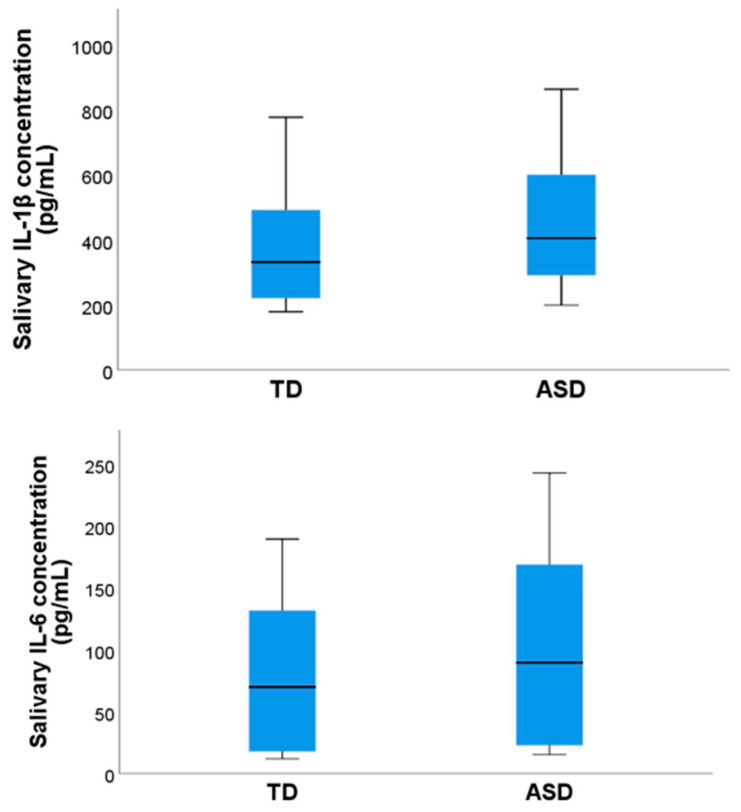
Salivary concentrations of IL-1β and IL-6 in the ASD and TD groups.

The mean concentration of IL-1β for the ASD group was 461.9 ± 44.5 pg/mL and 368.7 ± 38.9 pg/mL for the TD group (*p* = 0.12) (Figure 2).

**Figure 3:**
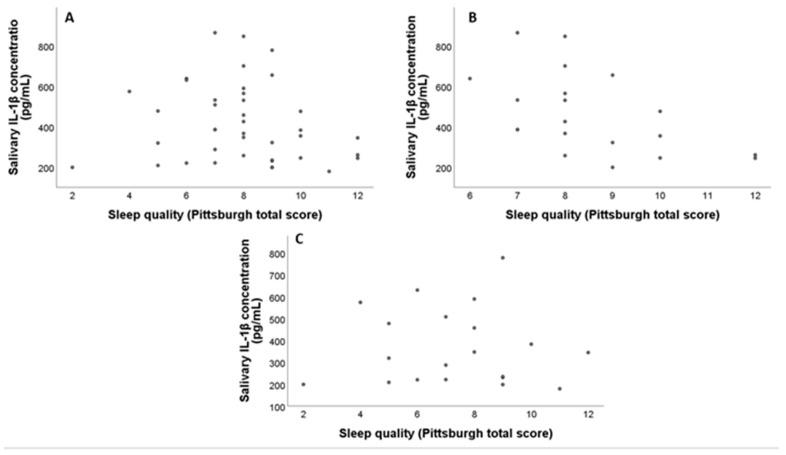
(**A**) Correlation between IL-1β concentration and sleep quality in the total sample; (**B**) correlation between IL-1β concentration and sleep quality in the ASD group; (**C**) correlation between IL-1β concentration and sleep quality in the TD group.

The mean value for the IL-6 concentrations in the ASD group was 99.6 ± 17.4 pg/mL, and in the TD group, it was 77.6 ± 13.5 pg/mL (*p* = 0.32) (Figure 2). No significant correlations were observed between IL-1β and IL-6 concentrations and age in the general sample (Rho = −0.05, *p* = 071; Rho = −0.26, *p* = 0.09). Furthermore, there were no significant correlations between IL-1β and IL-6 concentrations and age in the ASD group (Rho = −0.01, *p* = 0.93; Rho = −0.30, *p* = 0.19) or in the TD group (Rho = −0.10, *p* = 0.67; Rho = −0.25, *p* = 0.27). Significant correlations were found between the total PSQI score and IL-1β concentrations (Rho = −0.32, *p* = 0.04) (Figure 3A). However, no significant inverse correlations were found between the total PSQI score and the concentration of IL-6 (Rho = −0.06, *p* = 0.68, Spearman correlation) in the total sample. When the presence of correlations by groups was examined, for the ASD group, a significant inverse correlation was found between the PSQI total score and IL-1β concentration (Rho = −0.32, *p* = 0.04) (Figure 3B) but not between the PSQI total score and IL-6 concentrations (Rho = −0.11, *p* = 0.65).

No significant correlations were found in the TD group between the total PSQI score and the concentrations of IL-1β and IL-6 (Rho = −0.02, *p* = 0.92 and Rho = −0.005, *p* = 0.98, respectively, Spearman correlation) (Figure 3C).

Furthermore, no significant differences were observed for dichotomized sleep quality considering the cut-off point of >5 points indicative of poor sleep quality, with the concentrations of IL-6 and IL-1β in the TD group (*p* = 0.44, Mann–Whitney U test in all cases).

No significant differences were found between IL-1β and IL-6 concentrations and gender in the overall sample (*p* = 0.82 and *p* = 0.24, Mann–Whitney U test). Similarly, there were no significant differences between the concentrations of IL-1β and IL-6 by gender in the ASD group (*p* = 0.68 and *p* = 1.00) or in the TD group (*p* = 0.49 and *p* = 0.12, Mann–Whitney U test in all cases).

### 3.5. Multivariate Analyses

Linear regression analysis was performed to examine the relationship between sleep quality and several predictor variables, including age, sex, group (ASD vs. TD), and salivary IL-1β concentration. In the general sample, no significant associations were found between sleep quality and age, gender, or IL-1β concentrations in saliva, as shown in Table 2. However, there were significant associations between sleep quality in both groups (*p* = 0.03, OR = 0.36, 95% CI 0.14–2.93).

Additionally, a multivariate analysis was performed considering the same predictor variables but for the ASD group alone. Significant associations were found between sleep quality and IL-1β concentration (*p* = 0.01, OR = −0.53, 95% CI −0.008–0.001), while no significant associations with age or gender were found.

Similarly, no significant associations were found between sleep quality and variables such as age, gender, and IL-1β concentration in the TD group, as shown in Table 2.

## 4. Discussion

This study aimed to analyze the correlation between bad sleep quality in children/adolescents with ASD and inflammatory biomarkers, namely salivary IL-1 beta and IL-6, two cytokines previously found to be associated with poor sleep quality [27]. Measuring biomarkers through saliva is a more user-friendly method that increases the likelihood of cooperation from individuals with ASD [32,45].

In our study, we found that the entire sample (100%) of participants with ASD had poor sleep quality according to their parents’ reports, while only 25% of the participants in the control group slept poorly. The difference between the two groups was statistically significant for sleep latency, total sleep duration, sleep efficiency, and sleep interruption. The sleep quality in ASD has been reported previously [6,8,10,46,47].

The novel results of this study correlate reduced levels of IL-1β in saliva with poorer sleep quality using the PSQI questionnaire. We found no statistically significant correlation between sleep quality in participants with ASD and salivary levels of IL-1 beta.

The present study is the first to explore associations between pro-inflammatory cytokines measured in saliva and sleep quality in children/adolescents with ASD. In general, increased inflammation is believed to negatively affect sleep quality [48], but the fact that cytokines are pleiotropic and have physiological effects should also be taken into account. In addition to being a product of the peripheral immune system, IL-1β and its receptors are also expressed in the central nervous system (CNS) [49]. Likewise, not only does the CNS respond to changes in peripheral concentrations of these cytokines, but the cytokines are also produced and act in the CNS, thus supporting the idea that these cytokines have functions beyond immune responses [50], including sleep [51]. In this regard, IL-1β has been implicated in the regulation of sleep and non-rapid eye movement (NREM) sleep in several animal species [52]. Our results support the role of IL-1β in the regulation of physiological sleep because lower levels of salivary IL-1β were observed among participants with ASD who reported poorer sleep quality. We do not know the reason for the decrease in IL-1 beta in ASD and its association with sleep. One possibility may be stress in ASD that may stimulate the hypothalamic–pituitary–adrenocortical (HPA) axis and consequent cortisol and androgen production. The latter generates an anti-inflammatory response that downregulates IL-1β production [53]. As demonstrated by Corbett and co-workers, children with autism, but not neurotypical children, showed a more variable circadian rhythm as well as statistically significant elevations in cortisol following exposure to a novel, non-social stimulus [54], although the baseline level did not appear to be significantly different from that of the TD group [55] or was even lower than the TD baseline level [56]. An increase in adrenal androgen concentration has also been suggested to play a role in ASD symptoms [55] and the inhibition of the production of inflammatory cytokines (like Il-1 beta) [57]. The hyperactivity of the HPA axis could therefore be a biologically plausible mechanism for explaining the association between sleep disturbance and decreased IL-1β production in the ASD group. In the literature, both increases and decreases in IL-1 beta concentration have been related to poor sleep quality, suggesting the presence of non-linear associations with sleep quality. However, it is also possible that a more sustained activation of the immune system leads to increased IL-1β release and consequently increased sleep duration and efficiency. The association with IL-1β, sleep, and other inflammatory markers should therefore be studied further.

The limitation of this pilot study is the small sample size, despite its statistically significant results showing that people with ASD and poor sleep quality have elevated levels of IL-1β in saliva compared to the control group. The impact of drugs of different pharmacological classes and their combination should be explored in studies with larger sample sizes. Although we did not observe any significant effect on the study’s outcomes, we cannot rule out specific effects for some of these pharmacological groups as antidepressants have been shown to reduce IL-1β [58], while antipsychotics have no effects on IL-1β release [59] in other psychiatric disorders. In order to minimize the circadian fluctuation of cytokine production [24,60,61], we collected saliva samples in a narrow (2 h) window during the afternoon. We cannot assume that the same association between sleep quality and salivary IL-1 beta could be obtained during morning or at night, as the levels of this cytokine physiologically vary compared to the afternoon levels, nor can we assume that circadian variation in cytokine production could be the same in the ASD and TD groups.

The association between sleep, IL-1β, and nuclear or comorbid alterations in ASD should be explored in future studies, as this cytokine has a neuromodulating role in the central nervous system by intervening in the neuronal ion channels, participating in the neuronal excitability mechanism, and affecting brain neuroplasticity [62,63]. IL-1β also plays a role in memory consolidation and behavior [64,65]. Scientific evidence supports the relationship between sleep and learning and memory and suggests that sleep plays a key role in enhancing brain plasticity [66,67]. Lack of sufficient sleep time in terms of hours sleeping or poor sleep quality has been associated with a negative impact on cognitive functions (memory consolidation, learning acquisition, attention, executive functions, and brain maturation) [68,69,70] as well as on daytime behavior [71,72,73,74] in children with ASD.

## 5. Conclusions

To our knowledge, our study is the first to focus on the relationship between sleep quality and inflammatory cytokines in saliva in children/adolescents with ASD. The analysis of inflammatory cytokines in samples of saliva is easier in patients with ASD due to its ease of collection, and it also could act as a proxy of the systemic burden of peripheral inflammation in ASD, as reported in other populations.

The identification of a salivary biomarker, i.e., IL-1 beta, which is associated with poor sleep quality, could be tested further, not only to diagnose sleep problems but also to monitor the effects of interventions aimed to improve sleep quality in individuals with ASD.

## Figures and Tables

**Table 1:** Relationship of sleep quality between the ASD and control groups.

Pittsburgh Sleep Quality Index Subdomains	Comparison of Sleep Subdomains Between the ASD and Control Groups (*p* Value) (Mann–Whitney Test)
Subjective sleep quality	0.64
Sleep latency	0.02
Sleep duration	0.001
Sleep efficiency	0.001
Sleep disturbances	0.001
Use of sleep medications	0.04
Daytime dysfunction	0.00

**Table 2:** Relationship between sleep quality and variables, including age, sex, group (ASD vs. TD), and salivary IL-1β concentration.

Variable	Group	Standard B Coefficient	T	*p* Value	95% Confidence Interval: Lower Limit–Upper Limit
Age	Entire sample	−0.03	−0.93	0.83	−0.31 −0.25
Gender		−0.04	0.35	0.79	−2.17 1.68
Group		0.36	1.73	0.03	0.14 −2.93
Concentration of IL−1β		−0.21	−1.28	0.20	−0.006 −0.001
Age	ASD group	−0.24	−1.14	0.27	−0.37 −0.11
Gender		−0.10	−0.51	0.61	−2.19 −1.34
Concentration of IL−1β		−0.53	−2.63	0.01	−0.008 −0.001
Age	TD group	0.07	0.212	0.82	−0.58 −0.72
Gender		0.03	0.09	0.92	−4.03 −4.4
Concentration of IL−1β		−0.01	−0.07	0.94	−0.008 −0.007

## Data Availability

The data presented in this study are available upon request with a scientific purpose from the corresponding author.
